# Contrast-enhanced CT techniques and MRI perform equally well in arthritis imaging of the hand: a prospective diagnostic accuracy study

**DOI:** 10.1007/s00330-022-08744-0

**Published:** 2022-04-01

**Authors:** Sevtap Tugce Ulas, Katharina Ziegeler, Sophia-Theresa Richter, Sarah Ohrndorf, Robert Biesen, Fabian Proft, Denis Poddubnyy, Torsten Diekhoff

**Affiliations:** 1 Department of Radiology (CCM), Charité – Universitätsmedizin Berlin, Campus Mitte, Humboldt – Universität zu Berlin, Freie Universität Berlin, Charitéplatz 1, 10117 Berlin, Germany; 2 Department of Rheumatology, Charité – Universitätsmedizin Berlin, Campus Mitte, Humboldt – Universität zu Berlin, Freie Universität Berlin, Berlin, Germany; 3 Department of Gastroenterology, Infectious Diseases and Rheumatology, Charité – Universitätsmedizin Berlin, Campus Benjamin Franklin, Humboldt – Universität zu Berlin, Freie Universität Berlin, Berlin, Germany

**Keywords:** Computed tomography, Dual energy, Arthritis, rheumatoid, Magnetic resonance imaging, Ultrasound

## Abstract

**Objectives:**

To investigate the performance of dual-energy CT (DECT)-generated iodine maps (iMap) and CT subtraction (CT-S) in the detection of synovitis, tenosynovitis, and peritendonitis/paratenonitis compared to magnetic resonance imaging (MRI) using musculoskeletal ultrasound (MSUS) as standard of reference.

**Methods:**

This IRB-approved prospective study consecutively investigated patients with undifferentiated arthritis. All patients underwent MSUS, MRI and contrast-enhanced DECT of the hand; from the latter conventional CT-S, image-based iMap (iMap-I) and raw data-based iMap (iMap-RD) were reconstructed. CT and MRI datasets were scored for synovitis and tenosynovitis/paratenonitis applying the modified Rheumatoid Arthritis MRI Score (RAMRIS). Sensitivity, specificity, and diagnostic accuracy were calculated. Non-inferiority was tested using the one-tailed McNemar test. Correlation of sum scores was assessed using Pearson’s test. Interreader reliability was assessed using Cohen’s kappa.

**Results:**

Overall, 33 patients were included. MSUS was positive for synovitis and tenosynovitis/paratenonitis in 28 patients with a sum score of 6.91. Excellent correlation with MSUS was shown for CT-S (sum score 6.38; *r* = 0.91), iMap-RD (sum score 9.74; *r* = 0.82), MRI (sum score 12.70; *r* = 0.85), and iMap-I (sum score 6.94; *r* = 0.50). CT-S had the highest diagnostic accuracy of 83%, followed by iMap-I (78%), MRI (75%), and iMap-RD (74%). All modalities showed non-inferiority. Reader agreement was good for CT-S and MRI (*κ* = 0.62; 0.64) and fair for iMap-RD and iMap-I (*κ* = 0.31; 0.37).

**Conclusion:**

CT-S and iMap allow highly standardized arthritis imaging and are suitable for clinical practice. MSUS still has the highest availability for arthritis imaging and served as gold standard for this study.

**Key Points:**

*• CT subtraction, iodine map with dual-energy CT, and MRI showed non-inferiority to musculoskeletal ultrasound.*

*• MRI was the most sensitive but least specific imaging technique compared with CT subtraction and dual-energy CT.*

*• CT subtraction showed the best correlation with musculoskeletal ultrasound.*

**Supplementary Information:**

The online version contains supplementary material available at 10.1007/s00330-022-08744-0.

## Introduction

Early detection of synovitis and tenosynovitis is of major interest in the field of arthritis imaging [1], as early treatment is needed to prevent irreversible bone and joint destruction [2], and therefore contributes to the preservation of joint functionality and patients’ quality of life. It requires standardized and readily available imaging methods that enable reliable exclusion of differential diagnoses in order to justify the expensive anti-inflammatory therapy [3].

The current standard for arthritis diagnosis includes sensitive imaging methods such as musculoskeletal ultrasound (MSUS) and magnetic resonance imaging (MRI) with known advantages and disadvantages [1]. Having relatively low specificity, MRI is no longer the modality of first choice [4]. Its use is now mostly restricted to the quantification of inflammatory changes in the context of clinical trials [4]. MSUS is more readily available, can be used by clinicians during office hours, and provides higher spatial resolution than MRI [5–7]. For this reason, it has become the method of first choice for diagnosing early arthritis [4].

While conventional computed tomography (CT) has played a subordinate role in the diagnosis of arthritis so far [8], dual-energy CT (DECT) has developed into a valuable tool for the diagnosis of gouty arthritis. However, both techniques are limited in the assessment of active inflammation [8]. Nonetheless, recent developments such as CT subtraction (CT-S) and generation of iodine maps (iMap) from DECT can detect contrast agent uptake in active soft tissue inflammation in joints with sufficient diagnostic accuracy [9–11]. Additionally, CT has a decisive advantage in the detection of bone erosion, and is a fast and standardized imaging procedure suitable for follow-up and comfortable for the patients, while radiation exposure remains low when peripheral joints are imaged [12, 13].

As two different CT techniques are available for arthritis imaging, the objective of this study was to use both techniques, iMap and CT-S, to assess synovitis and tenosynovitis in patients with suspected rheumatoid arthritis of the hand and to compare their diagnostic accuracy with MRI using MSUS as standard of reference.

## Materials and methods

### Subjects

We prospectively included 38 consecutive patients with undifferentiated arthritis of the hand who presented to the rheumatology outpatient or inpatient clinic of our hospital between October 2018 and 2019 according to prior statistical sample size estimation. Patients with contraindications to MRI (claustrophobia or magnetic implants) and/or contrast agent (known allergic reactions, reduced kidney function (glomerular filtration rate < 60 ml/min/1.73 m^2^) and hyperthyroidism) were excluded.

The study was approved by the local ethics committee (EA4_005_18) and the Federal Office for Radiation Protection (Z5-22462/2-2019-039). All participants gave their written informed consent.

### Imaging procedures

All patients underwent DECT, MRI, and MSUS of the clinically dominant hand in random order. DECT and MRI were performed on the same day. MSUS was performed either on the same day or within 1 week before or after the other modalities. There was no change in treatment between the imaging examinations. The total examination time was approximately 4 min for DECT, 35 min for MRI, and 20 min for MSUS.

#### DECT

DECT was performed on a 320-row single-source CT scanner (Canon Aquilion ONE Vision, Canon Medical Systems) with sequential volume acquisition of two different energy datasets (135 and 80 kVp) before and 3 min after injection of contrast agent at a body weight–adjusted dose (1 ml/kg Ultravist 370 (Bayer)) according to the results of previous perfusion studies for optimal contrast of peripheral joints [14]. A *z*-axis coverage of 16 cm without table movement was used. Rotation time was 0.275 s. Primary reconstructions of the CT images from the datasets were calculated in 0.5 mm slice thickness in axial plane, and 0.5 mm MPRs in coronal and sagittal plane using a medium soft tissue kernel and 0.5-mm bone kernel. For image reading, iMap and CT-S were reconstructed with 3.0 mm slice thickness in axial, coronal, and sagittal orientations. Radiation exposure (estimated effective dose) was calculated using the overall dose-length product (DLP) and a conversion coefficient of 0.0008 [mSv × mGy^−1^ × cm^−1^].

#### MRI

MRI was performed on a 1.5-T scanner (Siemens MAGNETOM Avanto; Siemens Healthcare) using a fat-saturated contrast-enhanced T1-weighted sequence in coronal (slice thickness 3 mm, TR 719 ms, TE 11 ms, resolution matrix 512 × 256, flip angle 150°) and axial for imaging of the wrist (4 mm slice thickness, TR of 591 ms, TE of 15 ms, 320 × 192 resolution matrix, 90° flip angle) and metacarpophalangeal joints (4 mm slice thickness, TR of 507 ms, TE of 15 ms, 320 × 192 resolution matrix, 90° flip angle). Furthermore, the protocol included the following standard clinical pulse sequences for imaging inflammatory arthritis: coronal T1-weighted sequence and coronal short-tau inversion recovery (STIR) sequence. Contrast agent was administered at a body weight-adapted dose (0.2 ml/kg gadolinium-DOTA (Dotarem) or 0.1 ml/kg gadolinium-BTDO3A (Gadovist)).

#### MSUS

A senior radiologist (T.D.) and senior rheumatologist (S.O.), each with 10 years of experience in MSUS, performed the MSUS examinations using a high-frequency liner array transducer with 24 MHz (Aplio 500, Canon Medical Systems) according the recommendations of the European Society of Musculoskeletal Radiology (ESSR). Power Doppler US was used to detect increased perfusion in the target areas as an indicator of active inflammation.

### DECT image processing

#### iMap

iMap were calculated using the CT console (dual-energy image view and dual-energy raw data analysis, version 6, Canon Medical Systems). Material formulas were − 136/− 106 HU (80 kVp/135 kVp) for fat and 67/63 HU (80 kVp/135 kVp) for muscle soft tissue applying a gradient of 0.55 for iodine. Iodine maps were calculated using the image-based method (iMap-I) and the raw data-based method (iMap-RD).

#### CT subtraction

Pre- and postcontrast CT images at 80 kVp were postprocessed in a soft tissue kernel using a special software tool (SureSubtraction Ortho version 5, Canon Medical Systems) and were subtracted to obtain a bone-free color-coded CT-S.

### Image reading

After pseudonymizing of the images, two readers (K.Z. with 5 years and S.T.U. with 2 years of experience in musculokeletal imaging) scored the iMap-I, iMap-RD, CT-S, and contrast-enhanced T1-weighted images independently for synovitis, tenosynovitis, and peritendonitis. Scoring was performed using the Rheumatoid Arthritis Magnetic Resonance Imaging Score (RAMRIS) criteria [15] to assign synovitis scores of 0 to 3 separately for the wrist (radioulnar, radiocarpal, and intercarpal joint), metacarpophalangeal joints (MCP) II–V, and proximal interphalangeal joints (PIP) II–V. In addition, the flexor and extensor tendons (I–V) were separately scored for tenosynovitis and peritendonitis on a scale of 0 to 3 (modified RAMRIS) per finger. For simplicity, we here use the terms peritendonitis (used in DECT and MRI) and paratendonitis (used in MSUS [16]) interchangeably. The readers were blinded to all identifying and clinical information. First, the readers scored the MRI datasets, followed by CT-S, iMap-RD, and iMap-I with at least a 2-week interval between the sessions to prevent recall bias. If the readers disagreed on the presence or absence of synovitis, a consensus reading was performed. Furthermore, the number of bone marrow edema-positive patients was assessed separately on MRI. MSUS images were scored by the examiners themselves by transferring the abovementioned scoring system to MSUS [17].

### Statistical analysis

Statistical analysis was performed using GraphPad Prism (version 7 for MacOS, GraphPad Software). Scoring results were dichotomized into positive (RAMRIS synovitis > 0) versus negative for inflammation (RAMRIS synovitis = 0). On the patient level, the one-tailed McNemar test was performed to test for non-inferiority. Contingency tables were created to calculate sensitivity, specificity, positive predictive, and negative predictive value separately for iMap-I, iMap-RD, CT-S, and MRI using the Wilson/Brown method. Diagnostic accuracy was calculated separately on the patient level and joint level. Mean sum scores for each investigated imaging method were calculated using the average of the sum scores of both readers to assess their correlation with MSUS using Pearson’s *r* test. Agreement of the two readers was quantified by calculating Cohen’s kappa (*κ*) [18] for the presence or absence of inflammatory changes on the joint/tendon level. Agreement regarding extent of inflammation was assessed using intra-class correlation coefficients (ICC) with a two-way mixed model.

## Results

### Subjects

Thirty-eight patients were included in the study. In 3 patients, no contrast-enhanced MRI sequences were acquired due to extravasation. In 2 patients, DECT postprocessing failed because of technical issues.

**Fig. 1:**
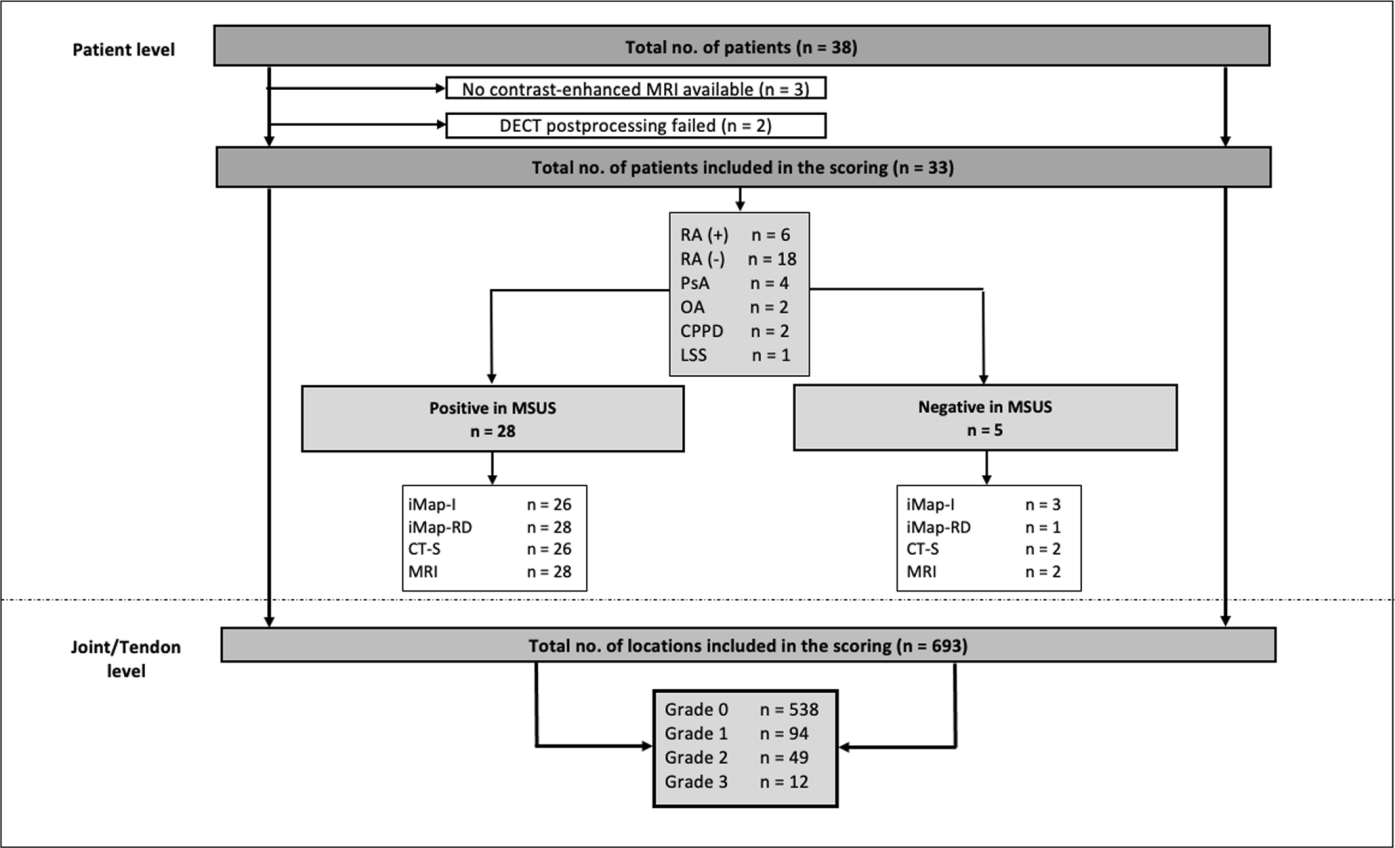
Flowchart of study inclusion and results of RAMRIS scoring. MSUS = musculoskeletal ultrasound, DECT = dual-energy CT, iMap-I = image-based iodine map DECT, iMap-RD = raw data-based iodine map DECT, CT-S = CT subtraction, MRI = magnetic resonance imaging, RA(+) = seropositive rheumatoid arthritis, RA(−) = seronegative rheumatoid arthritis, PsA = psoriatic arthritis, OA = osteoarthritis, CPPD = calcium pyrophosphate deposition disease, LSS = limited systemic sclerosis

A total of 33 patients (21 women) with a mean age of 55 years (SD 11.8, range 23–75 years) were available for statistical analysis. Mean C-reactive protein (CRP) was 18.3 mg/l (SD 30.3) with a mean duration of joint symptoms of 1.7 years (SD 3.8). Anti-citrullinated protein antibodies (ACPA) were positive ( > 20 IU/ml) in 6 patients (18.2%) and RF-IgM ( > 20 IU/ml) in 12 patients (36.4%). Twenty-four patients were finally diagnosed with rheumatoid arthritis (18 seronegative and 6 seropositive), four with psoriatic arthritis/peripheral spondyloarthritis, two with calcium-pyrophosphate-dehydrate deposition disease (CPPD), two with osteoarthritis, and one patient with limited systemic sclerosis. Fifteen patients were treatment-naïve, 7 patients were on csDMARD (6 glucocorticoids only), and 1 on bDMARD. Three patients received a combined therapy with glucocorticoids and csDMARD and one with csDMARD and bDMARD. A flowchart of patient inclusion is presented in Fig. 1.

Total DLP was 93.2 mGy * cm with an estimated effective dose of 0.075 mSv.

### Image reading and statistical analysis

**Fig. 2:**
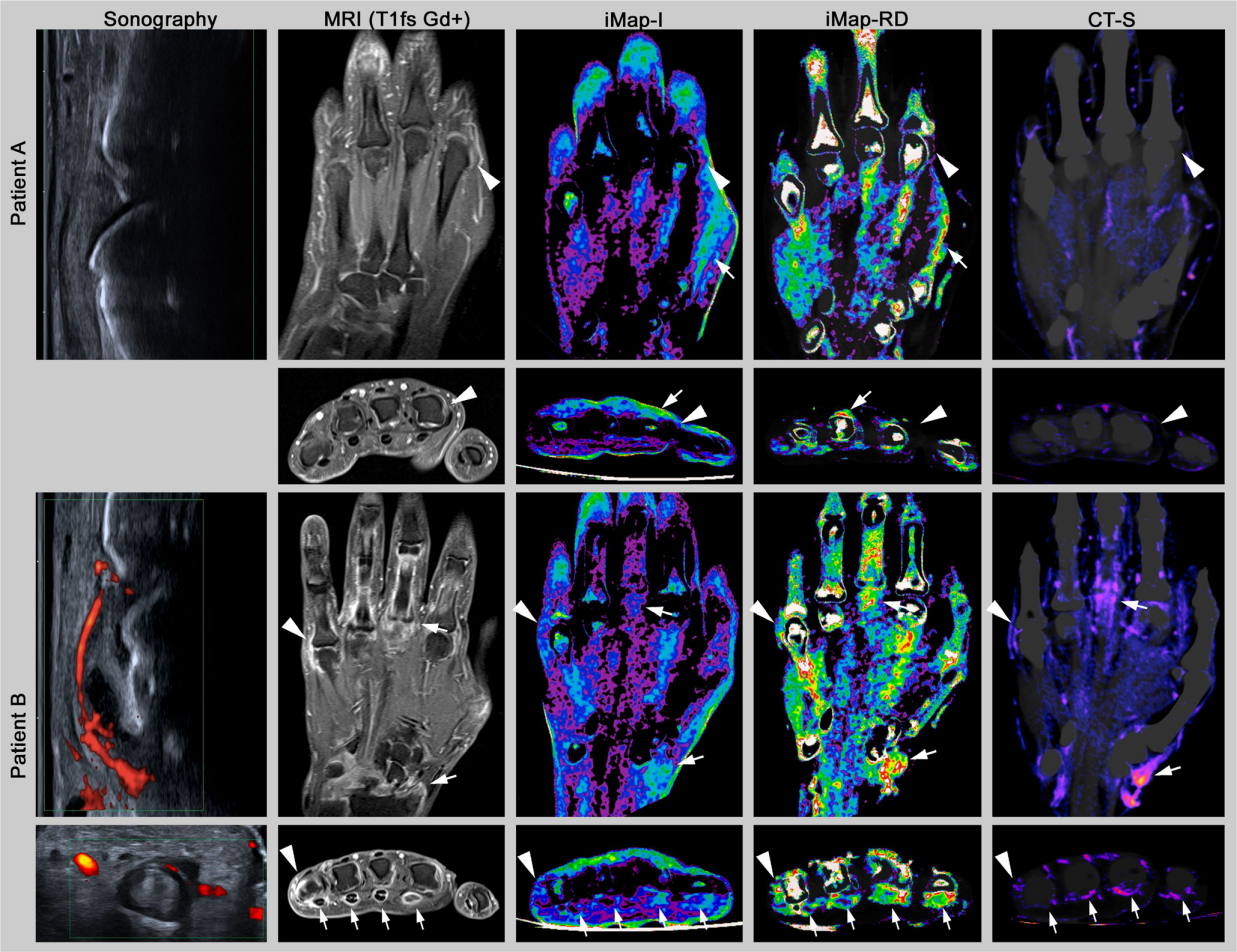
Imaging examples in coronal and axial orientation. MRI (T1fs Gd+) = fat-saturated contrast-enhanced T1-weighted sequence MRI; iMap-I = image data-based dual-energy CT iodine map; iMap-RD = raw data-based dual-energy CT iodine map; CT-S = CT subtraction. Patient A: a 50-year-old female patient with initial seronegative rheumatoid arthritis during therapy. No inflammatory joint changes were detected, e.g., in metacarpophalangeal joint II (MCP) (arrowhead). Note the partial volume effect at the bone and skin border (arrow). Patient B: a 55-year-old female patient with seronegative rheumatoid arthritis. There is severe synovitis in MCP V (arrowhead) and of the radiocarpal joint (arrow). Furthermore, severe tenosynovitis of the flexor tendons II–V was detected in all modalities (arrows), while iMap-I tends to underestimate the tenosynovitis III (arrow) and CT-S tenosynovitis V (arrow)

MSUS was positive for synovitis and/or tenosynovitis/peritendonitis in 28 patients (mean sum score 6.91 ± 7.76), iMap-I in 28 patients (mean sum score 6.94 ± 5.86), iMap-RD in 32 patients (mean sum score 9.74 ± 6.71), CT-S in 29 patients (mean sum score 6.38 ± 7.63), and MRI in 31 patients (mean sum score 12.70 ± 9.76). Bone marrow edema was detected in 8 patients. Imaging examples are shown in Fig. 2.

The results of the contingency table analysis are presented in Table 1. On the patient level, the highest sensitivity of 100% was calculated for iMap-RD and MRI. CT-S and iMap-I had 93% sensitivity. Diagnostic accuracy on the patient level was 91% for MRI, 88% for iMap-I and iMap-RD, and 85% for CT-S. On the joint/tendon level, the highest diagnostic accuracy of 83% was measured for CT-S. For iMap-RD diagnostic accuracy was 74%, for iMap-I 78% and for MRI 75%. McNemar’s test proved non-inferiority of all imaging modalities compared to MSUS: CT-S (*p* = 0.50), iMap-I (*p* = 0.31), iMap-RD (*p* = 0.07), and MRI (*p* = 0.12). The sum scores of CT-S and MSUS showed excellent correlation (*r* = 0.912, *p* < 0.0001), followed by MRI (*r* = 0.847, *p* < 0.0001) and iMap-RD (*r* = 0.816, *p* < 0.0001). Moderate correlation was shown for iMap-I (*r* = 0.504, *p* = 0.0028).

**Table 1:**
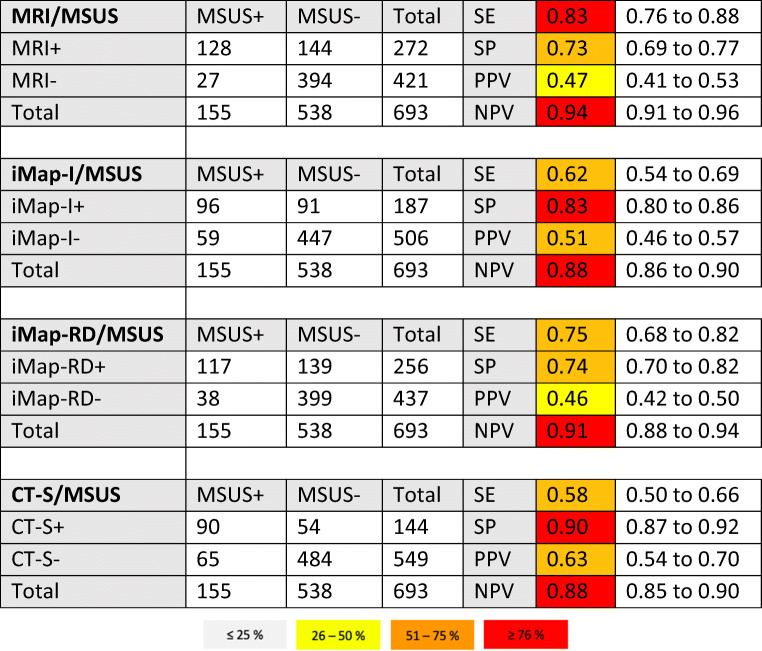
Results of contingency table analysis with 95% CI of the (modified) RAMRIS synovitis score. *MSUS* musculoskeletal ultrasound, *DECT* dual-energy CT, *iMap-I* image-based iodine map DECT, *iMap-RD* raw data-based iodine map DECT, *CT-S* CT subtraction, *MRI* magnetic resonance imaging, *SE* sensitivity, *SP* specificity, *PPV* positive predictive value, *NPV* negative predictive value

Agreement of the two readers was good for CT-S and MRI (Cohen’s *κ* = 0.62, 95% CI 0.54 to 0.69 and *κ* = 0.64, 95% CI 0.59 to 0.70). Only fair agreement was shown for iMap-RD and iMap-I (Cohen’s *κ* = 0.31, 95% CI–0.24 to 0.39 and *κ* = 0.37, 95% CI 0.29 to 0.44). Analysis of agreement regarding sum scores yielded ICCs of 0.923 (95%CI 0.844–0.962; *p* < 0.001) for MRI, 0.963 (95%CI 0.926–0.984; *p* < 0.001) for CT-S, 0.781 (95%CI 0.557–0.892; *p* < 0.001) for iMap-I, and 0.690 (95%CI 0.373–0.874; *p* < 0.001) for iMap-RD. CT-S was superior to iMap-I and iMap-RD (no overlap of 95%CIs); all other combinations showed no significant difference in agreement.

## Discussion

In this study, we investigated and compared different DECT reconstruction algorithms (iMap and CT-S) and MRI for the detection of synovitis and tenosynovitis/peritendonitis in patients with undifferentiated hand arthritis using MSUS as standard of reference. iMap-I and CT-S correctly identified 92.9% and iMap-RD and MRI identified 100.0% of patients with MSUS-proven arthritis; statistically, all imaging modalities were non-inferior to MSUS. An almost perfect correlation with MSUS was shown for CT-S (*r* = 0.912), while MRI (*r* = 0.847), iMap-RD (*r* = 0.816), and iMap-I (*r* = 0.504) had lower correlation. In a clinical setting, the choice of imaging modality for hand arthritis may depend on several infrastructural as well as patient-specific factors. MSUS is often the first-line imaging technique, as it is noninvasive and readily available [4]. MRI is a valid imaging modality for the context of clinical studies yet may be unsuitable in clinical practice due to its high cost and limited availability as well as its susceptibility for false-positive results for synovitis [4]. However, subclinical inflammation in the healthy population should not be ignored, as there is evidence of a link between subclinical inflammation in clinically suspected arthralgia (CSA) and the later development of arthritis [19]. Further studies are therefore needed here to investigate this causality. The total examination time of about 30 min is perceived as uncomfortable by some patients with joint pain [9]. CT, like MRI, is highly standardizable and additionally offers higher spatial resolution with more detailed depiction of erosions [20] as well as an unparalleled capacity for differential diagnosis (e.g., crystal deposition [21]) at the cost of radiation exposure. Using low-dose CT protocols, the effective dose is relatively low and comparable with a conventional chest x-ray; however, local skin dose can be quite high.

### DECT for detection of synovitis

Our results show good diagnostic accuracy for iMaps, comparable to the results reported by Fukuda et al [10]. In a further study of this group, iMaps were successfully used to quantify therapy response [22]. The high resolution of DECT images also helps in differentiating different inflammatory patterns [11]. Moreover, various DECT reconstructions can add diagnostic value by detecting and quantifying gouty tophi [23] and providing a decisive sign of bone marrow edema [24, 25]. The latter indicates severe inflammation and imminent erosion and cannot be depicted with MSUS [26].

### CT subtraction for the detection of synovitis

The availability of DECT technology has increased rapidly in recent years. However, CT scanners that do not have DECT capability can also be used for arthritis imaging using conventional CT-S [9, 27]. One advantage of the CT-S compared to iMaps is that it can also be created with a single-energy CT. In our study, CT-S was superior to iMap and MRI in correctly quantifying and identifying inflammation, i.e., it showed the highest diagnostic accuracy and best correlation with MSUS sum scores. When an appropriate low-dose CT protocol for peripheral joints is used, the total radiation exposure for acquisition of pre- and postcontrast images is comparable to that of an X-ray examination of the hand [9, 28]. In our study, iMaps and CT-S were not outperformed by MRI and, therefore, could be used as an alternative modality in clinical practice. A known limitation of CT-S is misregistration, but this can be overcome by using CT scans without table movement, as was done in our study. Nonetheless, CT techniques had higher specificity at the cost of sensitivity.

### Limitations

The study was deliberately designed as a proof-of-concept study with a small sample size. A major limitation of our study is that different readers assessed MSUS and the other cross-sectional imaging modalities. Furthermore, two different readers scored the MSUS. This may have had an impact on the overall assessment of inflammatory activity. While primary CT data showed 0.5 mm slice thickness, 3 mm multiplanar reconstructions were used for scoring to match the postcontrast T1-weighted imaging in coronal plane with 3.0 mm and in axial plane with 4.0 mm. However, in clinical practice, diagnostic with CT might profit from its higher spatial resolution. It was hypothesized that iMap-RD would have higher diagnostic accuracy than iMap-I based on the more accurate material separation but more artifacts near the skin, which may have had a detrimental effect on specificity. DECT also offers the possibility to assess bone marrow edema using the so-called virtual non-calcium reconstruction. However, due to low prevalence in our cohort (8/33), we did not further investigate its added value.

Contrast-enhanced CT and MRI performed equally well in comparison to MSUS. While MRI was the more sensitive imaging method, the CT techniques showed higher specificity and captured the extent of inflammation more accurately. In conjunction with its high spatial resolution and differential diagnostic capability, CT may evolve into a valuable alternative in early arthritis, especially in older patients or patients with contraindications to MRI. Further studies should investigate the impact of the different CT techniques on the diagnostic pathway and their role in treatment monitoring.

## Supplementary Information

ESM 1 (DOCX 33 kb)

## Abbreviations

ACPA: Anti-citrullinated protein antibodies
CPPD: Calcium-pyrophosphate-dehydrate deposition disease
CRP: C-reactive protein
CSA: Clinically suspected arthralgia
CT-S: CT subtraction
DECT: Dual-energy CT
DLP: Dose-length product
ESSR: European Society of Musculoskeletal Radiology
iMap: Iodine map
iMap-I: Image-based iodine map
iMap-RD: Raw data-based iodine map
LSS: Limited systemic sclerosis
MCP: Metacarpophalangeal joints
MSUS: Musculoskeletal ultrasound
OA: Osteoarthritis
PIP: Proximal interphalangeal joints
PsA: Psoriatic arthritis
RA(−): Seronegative rheumatoid arthritis
RA(+): Seropositive rheumatoid arthritis
RAMRIS: Rheumatoid Arthritis Magnetic Resonance Imaging Score
STIR: Short-tau inversion recovery sequence

## Declarations

### Guarantor

The scientific guarantor of this publication is Torsten Diekhoff, MD.

### Statistics and biometry

One of the authors has significant statistical expertise. No complex statistical methods were necessary for this paper.

### Methodology

- prospective
- cross-sectional study
- performed at one institution
